# Supplementary material for: Acculturation orientations affect the evolution of a multicultural society
Source: Nat Commun. 2018 Jan 4;9:58. doi: 10.1038/s41467-017-02513-0 (PMC5754364; doi:10.1038/s41467-017-02513-0)
Supplement: Supplementary file 1 — Supplementary Information [file 41467_2017_2513_MOESM1_ESM.pdf]

Supplementary Note 1: Generalized model with separate parameters controlling probabilities of immigration and interaction

The model presented in the main text makes the assumption that either a migration event or an interaction event occurs in each time step. This assumption reduces the number of model parameters, since the migration rate and the interaction rate are under the control of a single parameter: migration events occur with probability *m*, and, since an interaction event always occurs when a migration event does not occur, interaction events occur with probability 1–*m* . However, it is possible that our results are affected by this interdependence between both rates. To check this, we here present the results of a generalized version of the model, in which immigration and interaction events occur with independent probabilities in each time step. In this model variant, the probability that a migration event occurs is still denoted by *m*, but this parameter now has no control over the probability of an interaction event. Instead, the probability that an interaction event occurs in any given time step is under the control of a separate parameter, denoted as *i*. Hence, in the generalized model, it is no longer the case that either migration or interaction occurs in every time step; it is also possible that both events co-occur in the same time step (with probability *m**i*), or that neither event occurs in any given time step (with probability (1–*m*)*(1–*i*)).

Supplementary Fig. 1 gives an overview of model outcomes (frequency of the type I trait at the end of the simulations) for both variants of the model. For the original model, we present simulation outcomes for three values of *m.* For the generalized model, we present outcomes for nine combinations of *m* and *i*. For three of these combinations (shown in yellow), *i* is equal to 1–*m*, as is the case for the original model. Hence, for these three subgraphs, the probabilities of migration and interaction are the same as for the subgraphs for the original model in the same column (but in the original model, both events cannot co-occur in the same time step, and it is also not possible that neither event occurs). A comparison of the yellow subgraphs to the corresponding subgraphs of the original model shows that the assumption of independent probabilities of migration and interaction has no (or very minor) effects on the model outcomes.

Decoupling the migration rate and the interaction rate also allows us to assess their relative importance for the outcome of the model. When comparing the model outcomes within the generalized model in Supplementary Fig. 1, it is clear that the migration rate has a much stronger effect on model outcomes than the interaction rate (at least for the parameter values considered). Outcomes tend to be roughly similar across interaction rates when the migration rate is held constant (compare the subgraphs within the columns of Supplementary Fig. 1), whereas the opposite is not the case (compare the subgraphs within the rows of Supplementary Fig. 1). In Supplementary Fig. 1, we only show cases where the migration rate is much lower than the interaction rate. The reason is that a high migration rate (relative to the interaction rate) always leads to the fixation of the type I trait, regardless of the other parameters (Supplementary Fig. 1 shows that this is already almost the case for *m* = 0.25 and *i* = 0.75).

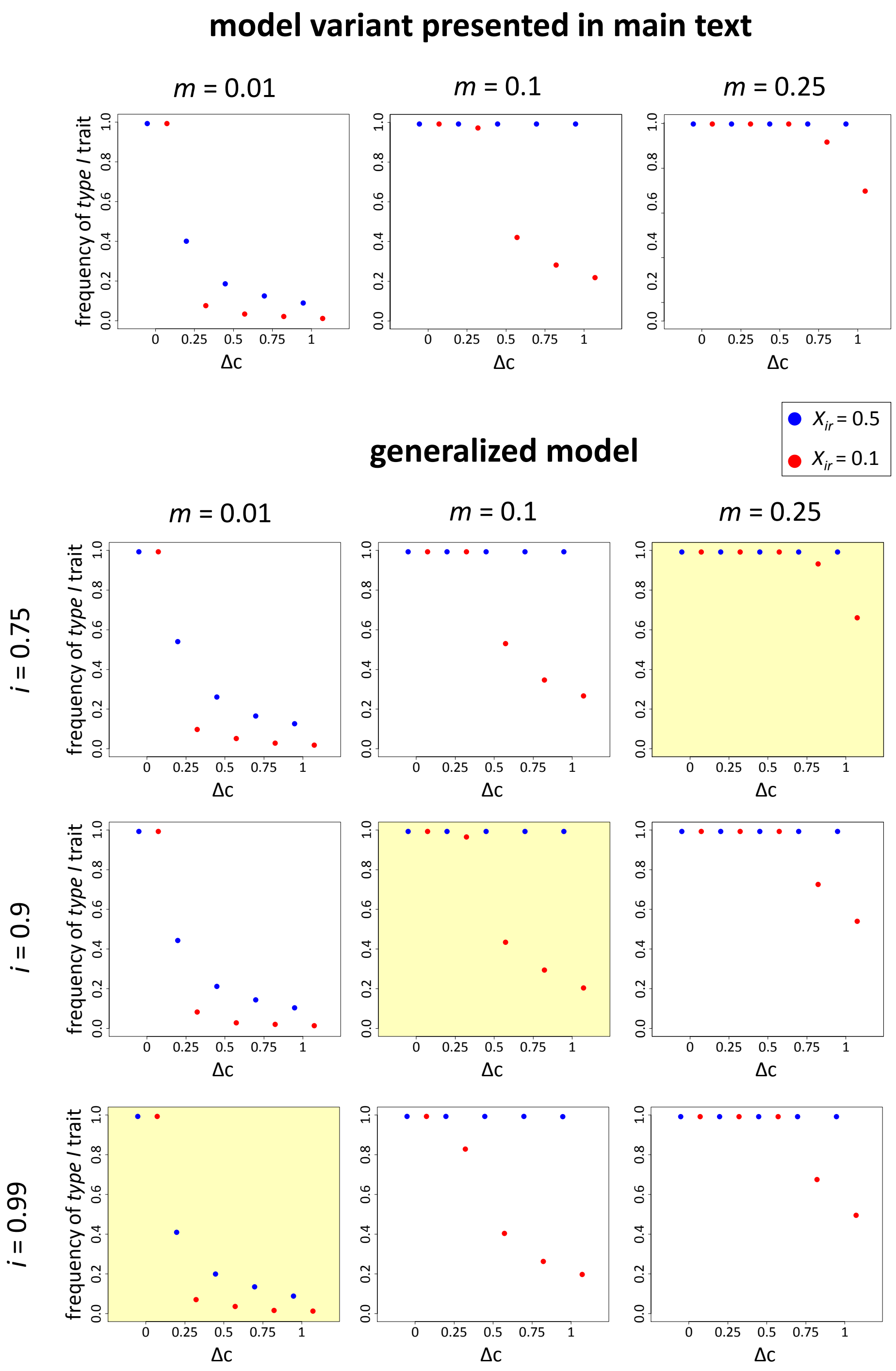

Supplementary Figure 1. Model outcomes for both model variants. Graphs show the frequency of the type I trait (which is initially associated with immigrants) at the end of the simulation, averaged over 10 replicate simulation runs per parameter combination. The top row shows outcomes for the original model variant (presented in the main text), for three values of the migration rate *m*. The bottom three rows show outcomes for the generalized model, for different combinations of the migration rate *m* and the interaction rate *i*. The subgraphs for the generalized model on the diagonal (in yellow) have the same probabilities of interaction and migration as the subgraph for the original model in the same column.
